# A cluster randomised controlled trial of a telephone-based intervention targeting the home food environment of preschoolers (The *Healthy Habits* Trial): the effect on parent fruit and vegetable consumption

**DOI:** 10.1186/s12966-014-0144-6

**Published:** 2014-12-24

**Authors:** Rebecca Wyse, Karen J Campbell, Leah Brennan, Luke Wolfenden

**Affiliations:** School of Medicine and Public Health, University of Newcastle, Newcastle, NSW Australia; Centre for Physical Activity & Nutrition Research, School of Exercise & Nutrition Sciences, Deakin University, Melbourne, VIC Australia; School of Psychology, Australian Catholic University, Melbourne, VIC Australia; Hunter Medical Research Institute (HMRI), Newcastle, NSW Australia; Hunter New England Population Health, Locked Bag 10, Wallsend, 2287 NSW Australia

**Keywords:** Fruit, Vegetable, Randomised controlled trial, Intervention, Telephone, Family, Home food environment, Preschool, Child

## Abstract

**Background:**

The home food environment is an important setting for the development of dietary patterns in childhood. Interventions that support parents to modify the home food environment for their children, however, may also improve parent diet. The purpose of this study was to assess the impact of a telephone-based intervention targeting the home food environment of preschool children on the fruit and vegetable consumption of parents.

**Methods:**

In 2010, 394 parents of 3–5 year–old children from 30 preschools in the Hunter region of Australia were recruited to this cluster randomised controlled trial and were randomly assigned to an intervention or control group. Intervention group parents received four weekly 30-minute telephone calls and written resources. The scripted calls focused on; fruit and vegetable availability and accessibility, parental role-modelling, and supportive home food routines. Two items from the Australian National Nutrition Survey were used to assess the average number of serves of fruit and vegetables consumed each day by parents at baseline, and 2-, 6-, 12-, and 18-months later, using generalised estimating equations (adjusted for baseline values and clustering by preschool) and an intention-to-treat-approach.

**Results:**

At each follow-up, vegetable consumption among intervention parents significantly exceeded that of controls. At 2-months the difference was 0.71 serves (95% CI: 0.58-0.85, p < 0.0001), and at 18-months the difference was 0.36 serves (95% CI: 0.10-0.61, p = 0.0067). Fruit consumption among intervention parents was found to significantly exceed consumption of control parents at the 2-,12- and 18-month follow-up, with the difference at 2-months being 0.26 serves (95% CI: 0.12-0.40, p = 0.0003), and 0.26 serves maintained at 18-months, (95% CI: 0.10-0.43, p = 0.0015).

**Conclusions:**

A four-contact telephone-based intervention that focuses on changing characteristics of preschoolers’ home food environment can increase parents’ fruit and vegetable consumption.

(ANZCTR12609000820202)

## Background

From a young age many children fail to meet minimum dietary guidelines for fruit and vegetable consumption [1,2]. Dietary patterns established in childhood track into adulthood [3], and insufficient childhood consumption of fruit and vegetables is linked to an increased risk of chronic disease in adults [4,5]. As such increasing the fruit and vegetable consumption of children represents a global health priority [6]. Ecological models suggest that the home food environment is an important setting for the development of dietary patterns in childhood [7]. Parents are gatekeepers to the home food environment [7,8] through: making foods available and accessible to children [9]: role modelling [10]; and establishing eating rules and encouraging specific eating behaviours [11]. In addition, parents’ own behaviours may be modified by the environment they establish for their children. As such, interventions that focus on changing the home food environment may impact the dietary patterns of parents as well as their children.

A recent systematic review of interventions to increase fruit and vegetable consumption among children aged 0–5 years [12] identified only one study attempting to change the home food environment. A four-contact, home-visiting program increased parent fruit and vegetable consumption, but did not change child consumption [13]. Since the review, the Infant Feeding Activity and Nutrition Trial (InFANT) showed that a parent intervention to improve infant diet, which also included strategies targeting some aspects of the home food environment, improved maternal diet quality [14]. The intervention was delivered via parenting groups over the first 18 months of the infants’ life, and focused on teaching parenting skills to support the development of positive diet behaviours in infancy. Furthermore, the *Healthy Habits* trial, demonstrated that a four-contact telephone-based intervention with parents that focused on creating a supportive home food environment could increase fruit and vegetable consumption among 3–5 year-old children [15]. After 12 months, the combined fruit and vegetable consumption of intervention children was significantly greater than consumption of control children [16]. However, after 18 months the difference between groups was no longer significant [16]. This intervention supported parents to make changes to their home food environment associated with higher fruit and vegetable consumption in children; increasing the availability and accessibility of fruit and vegetables in the home, increasing parental role-modelling, and introducing supportive home food routines [17-19].

There is a well-established literature regarding effective interventions to increase adult fruit and vegetable consumption, with systematic reviews finding evidence in favour of behavioural interventions [20,21], and specifically those utilising face-to-face nutrition education or counselling [22]. A review of environmental approaches to encourage healthy eating highlighted the potential for home-based environmental interventions, however the authors concluded there was a paucity of such interventions [23]. The current study attempts to address this gap in the literature and investigate the efficacy of a home food environment intervention on the dietary behaviours of parents. Specifically, this paper describes the changes in the average daily serves of fruit and of vegetables consumed by parents, at 2-, 6- 12- and 18-months, the secondary outcomes of the *Healthy Habits* trial. It was hypothesised that consumption among intervention parents would exceed that of controls at each follow-up time point.

## Methods

The trial was registered with the Australian New Zealand Clinical Trials Registry on Sept 21 2009 (ANZCTR12609000820202) and approved by the Hunter New England Health Human Research Ethics Committee. A detailed description of the methods employed in this cluster randomised controlled trial have been published elsewhere and are described briefly below [24]. Parents of 3–5 year-old children were recruited to the trial from 30 preschools in the Hunter region of NSW, Australia. Parents were allocated to an intervention (telephone support) or control condition (written information) using block randomisation in a 1:1 ratio based on the preschool of recruitment. Preschool randomisation was conducted by an independent statistician using a random number function in Microsoft Excel. Following collection of baseline data, parents were notified of their group allocation by letter.

### Intervention

The intervention, described in greater detail elsewhere [24], was developed in conjunction with a multi-disciplinary advisory group that included accredited practicing dietitians, psychologists specialising in parenting, and health promotion professionals. The intervention was conceptually guided by the model of family-based intervention used in the treatment and prevention of childhood obesity, as proposed by Golan and colleagues [25]. The model is grounded in socio-ecological theory and attempts to introduce new familial norms regarding healthy eating. The intervention was previously piloted in a small sample which demonstrated effectiveness, feasibility of delivery, and acceptability to parents [26]. The intervention was delivered through a series of four weekly 30-minute telephone calls. The calls were scripted and delivered by experienced telephone interviewers. The script content and homework activities were tailored based on parents’ responses and focused on; fruit and vegetable availability and accessibility, parental role-modelling, and supportive home food routines (e.g. children having set meal and snack times). Behaviour change techniques such as goal setting and role-modelling [27] were built into the script. The intervention also consisted of written resources. Intervention parents received the ‘Healthy Budget Bites’ cookbook, which was developed locally and was specifically designed to encourage healthy eating through the provision of simple, inexpensive recipes [28]. The *Healthy Habits* guidebook was designed to accompany each of the calls. It provided a summary of the content of each call, as well as factsheets with more detail about each of the included topics. There was dedicated space in the guidebook where participants were encouraged to record their goals and activities for each week. Intervention parents also received a pad of weekly meal planner templates. The intervention was delivered between April and December 2010. Approximately 6% of all intervention calls were monitored by members of the research team, with results indicating that 97% of key content areas of the script were covered, and in 80% of calls the telephone interviewers “rarely” deviated from the script [15]. Of the intervention parents, 181 (87%) completed all intervention calls [15], and the median number of call attempts per completed intervention call was three attempts for the Week 1 call, and two attempts for the calls in Weeks 2, 3 & 4.

### Control

Parents were mailed printed information about Australian dietary guidelines [29]. They received no further contact until the follow-up assessments.

### Data collection & measures

Telephone interviewers, blind to group allocation, collected data at baseline (from April to October 2010) and 2-, 6-, 12-, and 18-months later. Items from the Australian National Nutrition Survey [30] were used to assess the average daily serves of fruit and vegetables consumed by parents. *(How many serves of vegetables do you usually eat each day? One adult serve is a ½ cup of cooked vegetables or 1 cup of salad vegetables. How many serves of fruit do you usually eat each day? An adult serve is 1 medium piece or 2 small pieces of fruit or 1 cup of diced pieces).* A study of 1,598 Australian adults found these items were significantly associated with biomarkers of fruit and vegetable intake (alpha- and beta-carotene and red-cell folate) [31].

### Analysis

At each follow-up, generalised estimating equations were used to compare parents’ mean daily fruit and vegetable serves between groups. Fruit and vegetable outcomes were analysed separately. The analyses were adjusted for clustering by preschool and baseline values (i.e. baseline daily serves of vegetables and fruits respectively). An intention-to-treat approach was utilised, whereby participants were analysed based on the group to which they were originally allocated. All available data was used in the initial analysis. A sensitivity analysis was also conducted whereby baseline values were substituted for missing data (Baseline Observation Carried Forward). The trial was powered based upon the primary outcome (children’s fruit and vegetable consumption). However, using the same assumptions it was calculated that the sample would allow a between group detectable difference of 0.33 and 0.43 daily serves of fruit and vegetables respectively, with 80% power at the 0.05 significance level, after 18 months.

## Results

**Figure 1:**
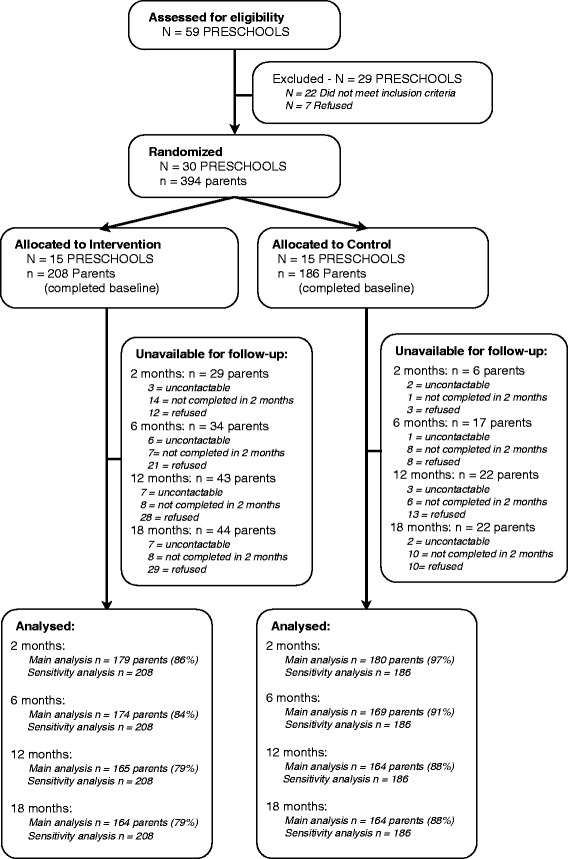
**CONSORT flowchart.**

A description of the study response rates and attrition is described in detail elsewhere [15,16]. Of the 394 parents recruited into the study 78% of those allocated to the intervention group and 88% of those allocated to the control group provided 18-month follow-up data (Figure 1).

Most parents who completed the baseline survey were female (96%), university educated (47%) with a household income over AUS$100,000 per year (41%); the average age was 35.4 years (SD = 5.4), and parents had an average of 2.3 children under 16 years (SD = 0.8) [32]. The participant characteristics by group are shown in Table 1.

**Table 1:** **Participant characteristics at baseline** [15]

**Participant characteristics**	**Intervention n = 208 mean (SD)/%**	**Control n = 186 mean (SD)/%**
Age – years	35.2 (5.6)	35.7 (5.0)
Gender - female	95.2%	96.8%
University Education	45.2%	49.5%
Annual household income (>AU$100,000)	42.4%	40.2%
Aboriginal or Torres Strait Islander	1.0%	3.2%
Number of children <16 y.o.	2.3 (0.8)	2.3 (0.7)

### Vegetable consumption

At each follow-up, intervention parents consumed significantly more vegetable serves than control parents (Table 2). Effect sizes ranged from 0.36 to 0.71 serves per day (approximately 27–53 grams) [33]. Although the sensitivity analysis attenuated the effect size (0.22-0.57 serves per day), the between-group difference remained statistically significant at each follow-up (Table 3).

**Table 2:** **Changes in participant consumption of vegetables and fruit (mean daily serves) using all available data**

	**Vegetable consumption (Mean daily serves)**						**Fruit consumption (Mean daily serves)**					
	**Control (SD)**	Control* (SD)	**Intervention (SD)**	Intervention* (SD)	Between group difference* (95% CI)	**P-value**	**Control (SD)**	Control** (SD)	**Intervention (SD)**	Intervention** (SD)	Between group difference ** (95% CI)	**P-value**
Baseline	3.05 (1.34)	-	3.25 (1.32)	-	-	-	1.76 (1.03)	-	1.83 (1.08)	-	-	-
2 mon	3.08 (1.26)	3.11 (0.68)	3.91 (1.41)	3.92 (0.64)	0.71 (0.58-0.85)	<0.0001	1.82 (1.04)	1.83 (0.69)	2.17 (1.08)	2.16 (0.74)	0.26 (0.12-0.40)	0.0003
6 mon	3.03 (1.51)	3.04 (0.68)	3.61 (1.40)	3.60 (0.63)	0.43 (0.19-0.68)	0.0005	1.95 (0.97)	1.96 (0.61)	2.04 (1.08)	2.04 (0.63)	0.06 (−0.14-0.26)	0.5405
12 mon	3.04 (1.37)	3.01 (0.80)	3.66 (1.77)	3.65 (0.74)	0.51 (0.30-0.73	<0.0001	1.81 (1.07)	1.81 (0.53)	2.08 (1.08)	2.09 (0.59)	0.22 (0.04-0.39)	0.0153
18 mon	3.06 (1.24)	3.06 (0.52)	3.53 (1.36)	3.53 (0.48)	0.36 (0.10-0.61)	0.0067	1.93 (1.03)	1.93 (0.54)	2.24 (1.07)	2.24 (0.57)	0.26 (0.10-0.43)	0.0015

*Adjusted for baseline value of daily vegetable serves and clustering by preschool.

**Adjusted for baseline value of daily fruit serves and clustering by preschool.

**Table 3:** **Changes in participant consumption of vegetables and fruit (mean daily serves): sensitivity analysis using baseline observation carried forward**

	**Vegetable consumption (Mean daily serves)**						**Fruit consumption (Mean daily serves)**					
	**Control (SD)**	Control* (SD)	**Intervention (SD)**	Intervention* (SD)	Between group difference * (95% CI)	**P-value**	**Control (SD)**	Control** (SD)	**Intervention (SD)**	Intervention** (SD)	Between group difference ** (95% CI)	**P-value**
Baseline	3.05 (1.34)	-	3.25 (1.32)	-	-	-	1.76 (1.03)	-	1.83 (1.08)	-	-	-
2 mon	3.09 (1.24)	3.11 (0.73)	3.82 (1.45)	3.79 (0.72)	0.57 (0.46-0.68)	<0.0001	1.81 (1.04)	1.81 (0.71)	2.08 (1.06)	2.08 (0.75)	0.21 (0.08-0.34)	0.0015
6 mon	3.11 (1.49)	3.10 (0.78)	3.56 (1.44)	3.54 (0.76)	0.32 (0.11-0.53)	0.0029	1.91 (1.01)	1.92 (0.70)	2.03 (1.10)	2.03 (0.73)	0.06 (−0.11-0.23)	0.4961
12 mon	3.05 (1.34)	3.03 (0.89)	3.56 (1.73)	3.55 (0.87)	0.39 (0.20-0.57)	<0.0001	1.79 (1.07)	1.79 (0.60)	1.99 (1.04)	2.00 (0.63)	0.16 (0.01-0.31	0.0382
18 mon	3.11 (1.24)	3.11 (0.69)	3.44 (1.41)	3.43 (0.68)	0.22 (0.01-0.43)	0.0374	1.89 (1.04)	1.89 (0.63)	2.13 (1.09)	2.13 (0.66)	0.19 (0.04-0.34)	0.0139

*Adjusted for baseline value of daily vegetable serves and clustering by preschool.

**Adjusted for baseline value of daily fruit serves and clustering by preschool.

### Fruit consumption

With the exception of 6-months, fruit consumption in the intervention group exceeded that of the control group at each follow-up, with effect sizes of 0.06-0.26 serves per day (9–39 grams) [33] (Table 2). The between-group difference remained significant when baseline values were substituted for missing values in the sensitivity analysis (0.06-0.21 serves per day) (Table 3).

## Discussion

Findings suggest that a telephone-based intervention focused on changing the home food environment of preschool children can increase their parents’ fruit and vegetable consumption, and that increases are sustained up to 18-months post-baseline. A systematic review found that primary prevention interventions targeting healthy adults increased combined fruit and vegetable consumption by 0.1-1.4 serves per day [22]. The increases in consumption in this trial ranged from 0.49 to 0.97 combined serves per day, with an average increase of 0.7 combined serves per day, and are consistent with these review findings, and with the average increase identified in a systematic review of behavioural interventions to increase fruit and vegetable consumption (0.6 serves per day) [20]. Furthermore, these findings are consistent with other telephone-delivered interventions to increase the fruit and vegetable consumption of adults [34]. Although these reviewed trials [35-37] were of a longer duration and included a greater number of calls than the current trial, telephone interventions consisting of fewer calls have also been successful in changing behavioural outcomes [38,39]. Most notably, a trial of an intervention consisting of written materials, a written plan, and two to three telephone calls (the most comparable intervention approach) increased combined fruit and vegetable consumption by 1.4 serves per day among primary care patients after 12 weeks [40]. The magnitude of the observed effect appears to have clinical significance, with meta-analyses conducted by the World Cancer Research Fund indicating that each 50 gram increase of vegetables per day reduced the risks of stomach, oesophageal, and mouth/pharynx/larynx cancers by 15%, 31% and 28% respectively [41]. This suggests that intervention approaches that target the home food environment may produce improvements in diet and reduce associated disease risks.

These significant findings are particularly noteworthy given these represent secondary trial outcomes, with the primary intervention aim being to increase preschoolers’ fruit and vegetable consumption. In fact, the changes in secondary trial outcomes were sustained for longer than the primary outcome (child fruit and vegetable consumption) [16]. This may be in part due to the intervention being delivered directly to parents rather than children, and accords with the theoretical underpinnings of the intervention that changes in familial norms and behaviours are antecedent to behaviour change for children [25]. This is most clearly illustrated through the intervention strategy of role-modelling, which directly relies on changes in parents’ consumption to facilitate changes in children’s consumption. Furthermore, the home food environment strategies that were targeted as part of the intervention required greater input from parents. For example, although increasing the availability of fruit and vegetables in the home is associated with higher consumption among both adults and children [17-19,42], adults are required to more actively respond to this strategy through making changes in their food purchasing and food preparation behaviours. Findings from child obesity treatment studies suggest that treating parents alone may be more effective than treating the parent and child together [43]. Although results from the *Healthy Habits* trial suggest dietary interventions involving parents can be effective [15,16], it is recommended future trials of dietary interventions investigate the relative effectiveness of strategies targeting parents-alone versus parents and children combined.

The non-significant finding for fruit consumption at 6-months reflects a slight increase in consumption of controls coinciding with a slight decrease in consumption of intervention parents. The increase in fruit consumption in the control group most likely reflects increases in the seasonal availability of fruits over the Australian summer period. This argument is strengthened by the similarly elevated fruit consumption among controls at the 18-month follow-up (i.e. the summer period the following year). The decrease in fruit consumption in the intervention group may reflect the typical attenuation of effect size over time [44]. Strategies that help maintain intervention effects are important to maximise the long-term benefits of dietary interventions. The results of this trial suggest that approximately 4 to 5 months post-intervention may be a critical point for the delivery of intervention maintenance strategies. However, further research is warranted.

The trial findings should be considered in conjunction with the limitations and strengths of this study. Strengths included the randomised controlled design, and standardised delivery of intervention scripts and data collection surveys. Use of self-reported, brief dietary measures may not represent optimal assessment of dietary intake and represent a limitation of the trial. More accurate assessments may result from alternative assessment methods, such as food records [45].

It is recommended that future trials investigate whether changes to the home food environment mediate changes in the fruit and vegetable consumption of children and their parents. A recent related study demonstrated that changes in child consumption of non-core foods were mediated by changes in the home food environment [46]. Identification of mediators of fruit and vegetable consumption could facilitate intervention streamlining. Beyond the cost efficiency afforded by telephone-delivery [47], this trial provides preliminary evidence of an additional efficiency; simultaneous increases in the fruit and vegetable consumption of preschool children [15,16] and their parents. Interventions targeting characteristics of the home food environment therefore appear to have substantial public health utility.

## Conclusion

A four-contact telephone-based intervention that focuses on changing characteristics of preschoolers’ home food environment can increase parents’ fruit and vegetable consumption. These results could inform the development of public health nutrition interventions attempting to improve the diet of preschoolers and their parents.

## Abbreviations

InFANT: Infant feeding activity and nutrition trial
SD: Standard deviation
95% CI: 95% Confidence interval
